# Supplementary material for: Decomposition of Glucose-Sensitive Layer-by-Layer Films Using Hemin, DNA, and Glucose Oxidase
Source: Polymers (Basel). 2020 Feb 4;12(2):319. doi: 10.3390/polym12020319 (PMC7077378; doi:10.3390/polym12020319)
Supplement: Supplementary file 1 [file polymers-12-00319-s001.pdf]

Supporting Information

Decomposition of Glucose-Sensitive Layer-by-Layer Films Using Hemin, DNA, and Glucose Oxidase

Kentaro Yoshida [1,*], Yu Kashimura [1], Toshio Kamijo [2], Tetsuya Ono [1], Takenori Dairaku [1], Takaya Sato [2], Yoshitomo Kashiwagi [1] and Katsuhiko Sato [2,3]

[1] School of Pharmaceutical Sciences, Ohu University 31-1 Misumido, Tomita-machi, Koriyama, Fukushima 963-8611, Japan; 714024@ohu-u.jp (Y.K.); t-ono@pha.ohu-u.ac.jp (T.O.); t-dairaku@pha.ohu-u.ac.jp (T.D.); y-kashiwagi@pha.ohu-u.ac.jp (Y.K.)

[2] Department of Creative Engineering, National Institute of Technology, Tsuruoka College, 104 Sawada, Inooka, Tsuruoka 997-8511, Japan; kamijo@tsuruoka-nct.ac.jp (T.K.); takayasa@tsuruoka-nct.ac.jp (T.S.)

[3] Graduate School of Pharmaceutical Sciences, Tohoku University, 6-3 Aoba, Aramaki, Aoba-ku, Sendai 980-8578, Japan; satok@m.tohoku.ac.jp

* Correspondence: k-yoshida@pha.ohu-u.ac.jp; Tel.: +81-24-932-8931

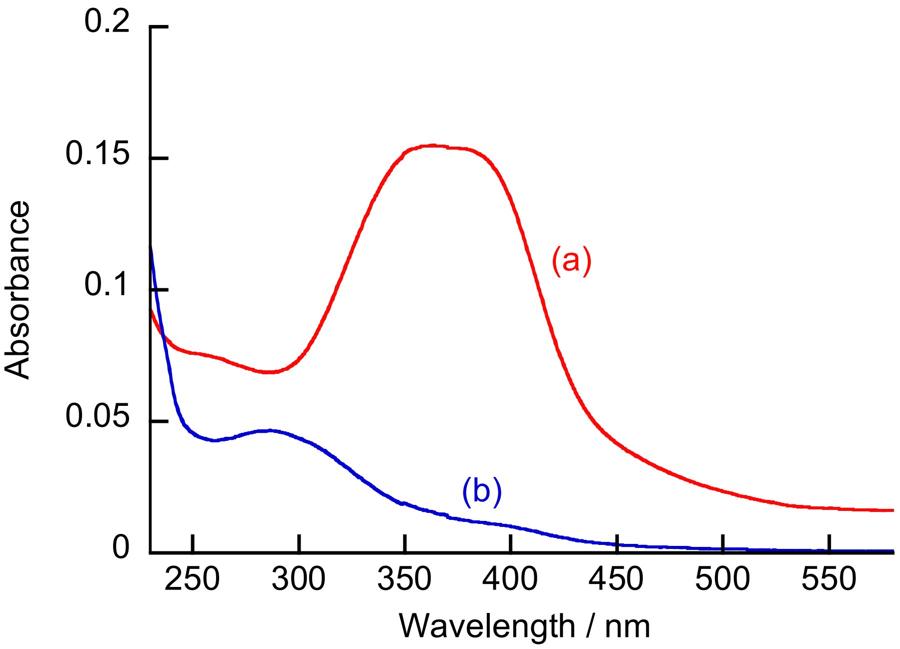

Figure S1. UV-vis absorption spectra for 30 μg/mL hemin solution (**a**) before and (**b**) after treated with 100 mM H_2O_2 (pH 7.4) for 1 h. The remaining H_2O_2 was consumed by dropping 10 μL of 0.1 mg/mL catalase into the test solution (2 mL) and letting it stand for 24 h.

Table S1. Absorbance difference in hydrogen peroxide treatment.

	**Δ Absorbance at 390 nm**	**Δ Absorbance at 260 nm**
30 μg/mL hemin solution	−0.143 ± 0.050	−0.033 ± 0.022
(H-PEI/DNA+GOx)$_5$ film	−0.127 ± 0.012	−0.094 ± 0.022

Absorbance difference for 30 μg/mL hemin solution and (H-PEI/DNA + GOx)$_5$ film treated with 100 mM H_2O_2 (pH 7.4) for 1 h. The remaining H_2O_2 was consumed by dropping 10 μL of 0.1 mg/mL catalase into the test solution (2 mL) and letting it stand for 24 h. The (H-PEI/DNA + GOx)$_5$ films treated with H_2O_2 was immersed in buffer for 5 min.

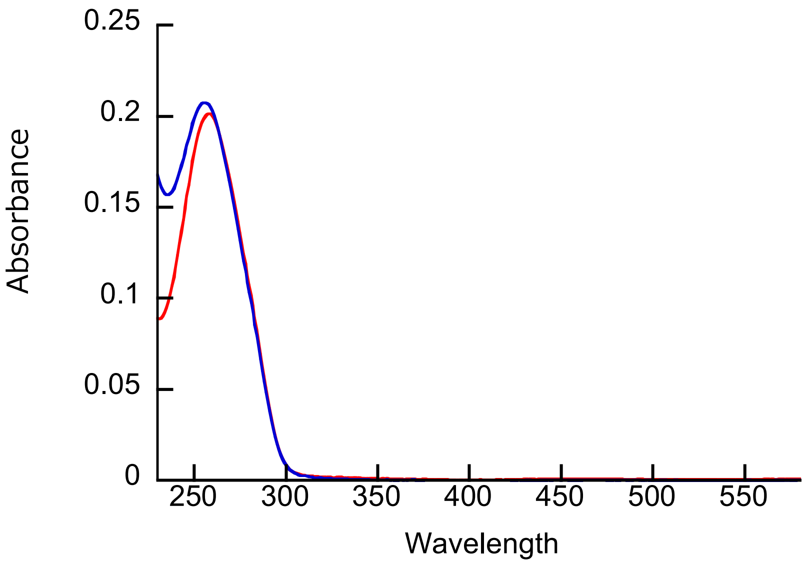

Figure S2. UV-vis absorption spectra for 13.6 μg/mL DNA solution (red) before and (blue) after treated with 100 mM H_2O_2 (pH 7.4) for 1 h. The remaining H_2O_2 was consumed by dropping 10 μL of 0.1 mg/mL catalase into the test solution (2 mL) and letting it stand for 24 h.

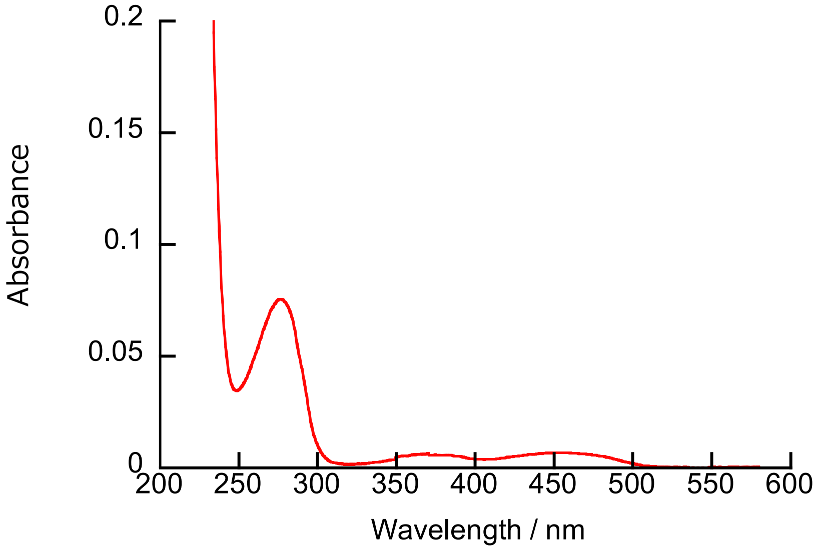

Figure S3. UV-vis absorption spectra for 110 μg/mL GOx solution (pH 7.4).
